# The Risk of Thrombosis Around Pregnancy: Where Do We Stand?

**DOI:** 10.3389/fcvm.2022.901869

**Published:** 2022-05-26

**Authors:** Jean-Christophe Gris, Florence Guillotin, Mathias Chéa, Chloé Bourguignon, Sylvie Bouvier

**Affiliations:** ^1^Department of Hematology, Nîmes University Hospital, Nîmes, France; ^2^Department of Hematology, Faculty of Pharmaceutical and Biological Sciences, Montpellier University, Montpellier, France; ^3^UMR UA11 INSERM-Montpellier University IDESP, Montpellier, France; ^4^Department of Obstetrics and Gynecology, First Ivan Setchenov Medical University, Moscow, Russia

**Keywords:** pregnancy, puerperium, thrombosis, risk factor, prophylaxis

## Abstract

Pregnancy and puerperium increase the relative risk of venous thromboembolism (VTE) and the absolute risk remains low, around 1 per 1,000, with induced mortality of around 1 per 100,000. Analysis of large databases has helped specify the modes of presentation and risk factors (RF) whose impact is greater after than before childbirth, since VTE during pregnancy and post-partum obey different RFs. The evolution of the population concerned (mostly women over 35, obese, of multi-ethnicity undergoing medically assisted reproduction) affects the frequency of these RFs. Pulmonary embolism (PE) is over-represented after childbirth, but 30% of PE in pregnancy occurs without any RFs. Recommendations for prevention, mainly from expert groups, are heterogeneous and often discordant. Low molecular weight heparins (LMWH) are the mainstay of pharmacological thromboprophylaxis, in a field where randomized controlled studies are definitely lacking. VTE risk assessment in pregnancy must be systematic and repetitive. Risk assessment methods and scores are beginning to emerge to guide thromboprophylaxis and should be used more systematically. In the future, analyzing observational data from huge, nationwide registries and prospective cluster clinical trials may bring to light clinically relevant outcomes likely to feed comprehensive guidelines.

## Introduction

Although the epidemiology and risk factors of venous thromboembolism (VTE) associated with pregnancy and puerperium have become more familiar, its efficient, medically-economical, individual prevention remains unclear.

**Figure 1:**
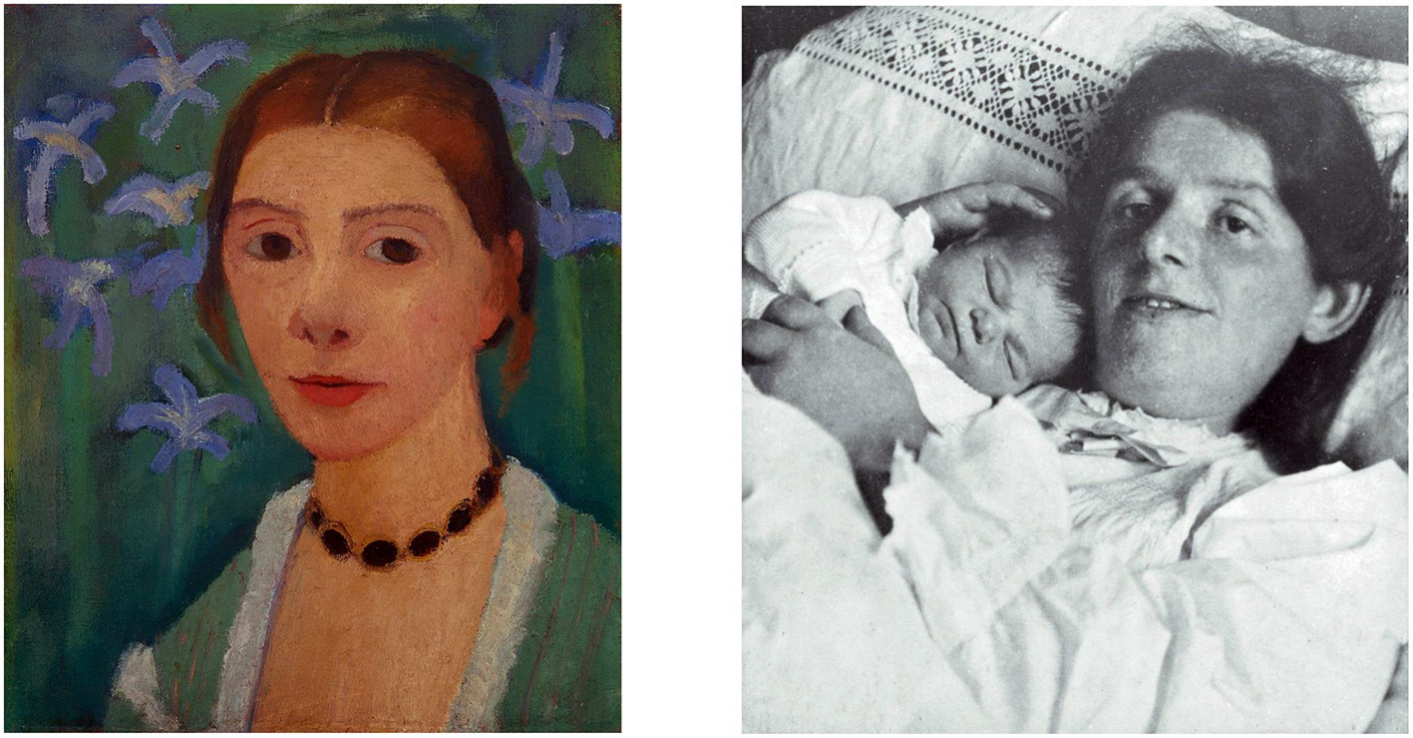
Paula Becker (February 8, 1876–November 20, 1907), German painter, one of the most important representatives of early expressionism. Self-portrait and photography after giving birth.

Pregnancy, and the 3 months following childbirth, increase the average relative risk of VTE by 4 to 5 (1, 2). The absolute risk of VTE during pregnancy and puerperium, estimated per thousand deliveries, is however limited: 1.4 (1.0–1.8), divided into 1.1 (1.0–1.3) for deep vein thrombosis (DVT) and 0.3 (0.3–0.4) for pulmonary embolism (PE) (1, 2). Induced mortality ranges from 0.8 to 1.9 per 100,000 deliveries, or 8–10% of maternal mortality in industrialized countries (Figure 1; see Author's note at the end).

DVT mainly affects the left lower limb (88 vs. 55%), is more often proximal (iliofemoral axis: 72 vs. 9%; compression effect of pelvic engagement of the fetal head at the end of pregnancy) and more often generates a post-thrombotic syndrome (3).

The risk increases with the progression of pregnancy, peaks after delivery and normalizes 12 weeks later. Although two thirds of events occur before delivery and half during the third trimester, a quarter are diagnosed in the 3 weeks following delivery: postpartum is characterized by the highest daily incidence (1).

PEs occur mainly after delivery (60% of cases in the 2001–2006 Australian series involving over 500,000 pregnancies: 1 per 2,220 deliveries) (4) with 2% mortality and French data from 2013 show a PE/VTE ratio over 3 times higher in weeks 2–7 after delivery than during pregnancy (5).

## Risk Factors for VTE Around Pregnancy

In 2008, using a case-control approach on hospital enrolments, the Norwegian Jacobsen et al. (6) was the first to show that clinical risk factors (RFs) were different before and after delivery. These risk factors can be classified as pre-existing, intercurrent-transitory and pregnancy-specific. Adding them together qualifies the individual risk, which may therefore change and require regular assessment. With regards to ante-partum RFs, immobilization -in relation with multiple pregnancy, or a diagnosis of placenta praevia and premature rupture of membranes-, defined as a strict bed rest 1 week or more prior to delivery or to the diagnosis of VTE, was associated with the highest adjusted risks, with a striking multiplied risk effect in women with a high body mass index (BMI), defined as higher than 25 kg.m^−2^ (6). The same was observed for postnatal VTE, with a significant effect of antepartum immobilization, and again high BMI in combination with antepartum bed rest being associated with the stronger risk for postnatal VTE (6). In both cases, the VTE risk associated with immobilization was stronger than the one associated with overweight (6).

The 1995–2009 UK cohort, analyzing over 280,000 women and 375,000 pregnancies (7), confirmed that the RFs for VTE before and after delivery differ, and the risk per patient-year is 4 times higher after delivery than before. Significant RFs only have a very modest effect on the incidence of VTE during pregnancy (Table 1A) and are present in 70% of PEs occurring at that time. Significant RFs for the post-partum period have a more sustained absolute effect but the average effect is <2, or 3% at the most. The same team also showed that the duration of risk associated with post-partum RFs was variable: 3 weeks for preterm birth and hemorrhage in labor and 6 weeks for Cesarean section, pre-eclampsia, obesity and acute infections (8). VTE risk factors during pregnancy have been recently reviewed by the Working Group in Women's Health of the German Society of Thrombosis and Haemostasis (9) (Table 1B), showing the striking impact of a personal history of VTE among preexisting RFS, of ovarian hyperstimulation syndrome and of the multiplicative interaction between antepartum immobilization and pre-pregnancy overweight among transient risk factors, and to a lesser extent of transfusion among pregnancy-associated risk factors.

Table 1Risk factors (RFs) for venous thromboembolism (VTE) around pregnancy.

**Table:** 

**(A) RFs identified in the UK population cohort, 1995-2009** **(****7****)**.		
	Mean variation in the relative risk*	Absolute risk, %: mean value (upper value)**
Antepartum VTE		
Medical comorbidities
Urinary infections	+80%	0.11 (0.16)
Varicose veins	+120%	0.16 (0.21)
Inflammatory bowel disease	+250%	0.22 (0.75)
Pre-existing diabetes mellitus	+250%	0.21 (0.42)
PostpartumVTE, 6 weeks postpartum		
Body mass index > 30 kg.m^−2^	+245%	0.70 (1.17)
Medical comorbidities
Varicose veins	+290%	1.00 (1.48)
Inflammatory bowel disease	+300%	1.14 (2.73)
Cardiac disease	+430%	1.69 (7.75)
Pregnancy complications
Cesarean delivery	+90%	0.48 (0.59)
Premature childbirth	+130%	0.64 (0.84)
Obstetrical hemorrhage	+150%	0.72 (1.34)
Stillbirth	+300%	1.83 (4.10)

*Reference: criterion-free pregnant woman.

**For a hundred 9-month-long pregnancies meeting the criterion.

**Table:** 

**(B) Classification of RFs during pregnancy with their corresponding adjusted odds ratios (95% confidence intervals) (OR, 95%CI) in the review performed by the Working Group in Women's Health of the German Society of Thrombosis and Haemostasis (GTH), 2020** **(****8****)**.					
**Preexisting RFs**	**OR, 95%CI**	**Transient RFs**	**OR, 95%CI**	**Pregnancy-associated RFs**	**OR, 95%CI**
Parity **>** 3	1.0 (0.6–1.8)	*In vitro* fertilization	2.7 (2.1–3.6)	Weight gain > 21 kg	1.6 (1.1–2.6)
Age > 35 years	1.5 (1.1–2.2)	Ovarian hyperstimulation syndrome	87.3 (54–141)	Cesarean section	2.1 (1.8–2.4)
Smoking*	2.1 (1.3–3.4)			Multiple pregnancy	2.7 (1.6–4.5)
Familial VTE**	2.2 (1.9–2.6)	Antepartum immobilization^+^		Preterm delivery°	2.7 (2.0–6.6)
Anemia	2.6 (2.2–2.9)	If no overweight^++^	7.7 (3.2–19)	Preeclampsia	3.1 (1.8–5.3)
Varicose veins	2.7 (1.5–4.7)	If overweight^++^	62.3 (11.5–337)	Severe peripartum hemorrhage^°°^	4.1 (2.3–7.3)
Obesity***	4.4 (3.4–5.7)			Postpartum infection	4.1 (2.9–5.7)
Prior VTE	24.8 (17.1–36)			Stillbirth	6.2 (2.8–14.1)
				Transfusion	7.6 (6.2–9.4)

*Defined as 10-30 cigarettes per day prior to or during pregnancy.

**Family history of VTE in any relative.

***Defined as a body mass index value > 30 kg.m^−2^.

*^+^Defined as a strict bed rest > to 1 week*.

*^++^Defined as pre-pregnancy body mass index value > 25 kg.m^−2^*.

*°Defined as before 37 weeks*.

*^°°^Defined as > 1L of blood loss*.

Blondon's meta-analysis of the risk associated with Cesarean section certainly showed a four times higher increase in risk, and even more so in the event of urgent procedures but with an average of hardly 3 thrombotic events per 1,000 Cesareans (10).

The Australian group focusing on the risk factors for postpartum PE (4) identified planned Cesarean section (relative risk RR: 3.2), Cesarean section during labor (RR: 3.7), red blood cell transfusion (RR: 3.9), stillbirth (RR: 6.0), other transfusions and infusion of procoagulant fractions (RR: 8.2) and, finally, lupus (RR.8.8). However, in that setting, a relative risk of 6, means hardly one PE per 1,000 deliveries fulfilling the corresponding clinical criteria.

Moving from these data to prevention, first, it cannot target women who develop a pulmonary embolism during their pregnancy in the absence of any identifiable risk factor (30% of cases in the British group). Nor can it be directly applied to women with only one risk factor: an enormous prescribing effort would be required for prevention in the event of one single postpartum RF for VTE as identified by the UK group. For instance, based on an 80% efficacy of low-molecular weight heparins (LMWH), the number of women to treat during 6 weeks for avoiding one VTE event would be 1,598 in case of preeclampsia. The therapeutic intervention, in terms of the number of injections required to avoid a VTE event, is considerable (in the previous case of a woman with pre-eclampsia: 67,116 injections to avoid one VTE event) and thus of dubious medico-economic efficiency, with the risk of inducing a hemorrhagic becoming significant. The ideal solution would be to target only those women who have accumulated such a high risk of VTE that the absolute risk incurred exceeds the consensus threshold, outweighs the iatrogenic risks incurred and retains a medico-economic virtue.

Risk assessment is also carried out within a changing population, with more and more obese pregnant women, higher age of first pregnancy and more pregnant women over the age of 35, increasing use of medically-assisted procreation (MAP: *in vitro* fertilization and other methods and techniques based on the laboratory manipulation of reproductive cells; i.e., assisted reproduction techniques), more and more Cesarean deliveries and increasing multi-ethnicity. MAP is accompanied by an increased risk in the first trimester, mainly after ovarian hyperstimulation syndrome (11, 12), with an absolute risk of 1.7% and, in the USA, the risk of thrombosis during pregnancy is lower in patients of Asian origin and higher in Afro-American women (13). One large study conducted at a hospital in Dublin on 21,000 deliveries (14) showed that age over 35 years, overweight or Cesarean section were present in one third of the women for each of the three criteria, with three quarters of them having at least one post-partum risk of VTE, with the application of international recommendations leading to prevention measures being prescribed for 7 to 37% of cases!

In women with a personal history of VTE, pregnancy also carries a risk of VTE recurrence. The RIETE registry restricted to women affected by VTE during pregnancy showed a 3.3% (1.5–5%) risk of recurrence at 2 years i.e., 2.3 recurrences per 100 patient-years (15). In the 2002 Vienna study, a new pregnancy increased that risk (RR: 3.5 (1.5–7.8) (16). The study by Brill-Edwards et al. (17) on a limited group of patients, suggests that the risk of recurrence during pregnancy was low (0% although the maximum calculated was 8%) if the first event was caused by a transient RF and if thrombophilia screening was negative.

The question of risk of a first VTE event in pregnancy in a patient with previously asymptomatic thrombophilia is frequently raised. The latest Bayesian meta-analysis identifies high-risk traits (18). Antithrombin deficiency induces an absolute risk in pregnancy of 7.3% (1.8–15.6%) and of 11.1% (3.7–21%) during puerperium. For protein C deficiency, the risk is 3.2% (0.6–8.2%) in pregnancy and 5.4% (0.9–13.8%) in the postpartum period. For protein S deficiency: 0.9% (0.0–3.7%) in pregnancy and 4.2% (0.7–9.4%) after delivery. For homozygous factor V Leiden polymorphism, it is 2.8% (0.0–8.6%) in pregnancy and 2.8% (0.0–8.8%) in puerperium. On the other hand, the cumulative risk (pregnancy + post-partum) of heterozygous V Leiden, of heterozygous FII 20210A and of their combination are all <3% (18) and we will see that this absolute risk threshold is proposed to justify thromboprophylaxis during postpartum.

## The Precarious Pathway from Risk Factors to Thromboprophylaxis

It is not easy to move on from an epidemiological approach describing the RFs for VTE during pregnancy and postpartum to an informed, balanced therapeutic proposal for prophylaxis which is both medically and economically acceptable. No placebo-controlled trials can be used to consolidate one particular approach. A large number of expert recommendations are available but these often disagree and are not regularly updated. Critical analysis using the AGREE II instrumental score (19) highlights their variable quality, inconsistencies, questionable methodologies and insufficient independence from the drug industry. One remarkable American single-center study (20) assessed the percentages of post-partum pharmacological thromboprophylaxis that would result from applying the recommendations of the American College of Obstetricians and Gynecologists (ACOG), the American College of Chest Physicians (ACCP) and the Royal College of Obstetricians and Gynecologists (RCOG) on 293 Cesarean section cases. The values obtained vary significantly (1, 35, and 85%, respectively).

The absolute thrombotic risk threshold justifying thromboprophylaxis has not been definitively decided.

During pregnancy itself, the available recommendations are still evasive.

During the postpartum period, extrapolating from general surgery patients, the ACCP experts (21) first evaluated the balance of desirable and undesirable consequences of a LMWH-prophylactic treatment, second focused on pregnancy-specific considerations then defined an absolute risk of VTE suggesting prophylaxis. The postpartum risk of major bleeding was estimated to be 0.3% (0–1%). The case-fatality rate of major VTE was estimated 1% (0.9–2.2%), the one of major bleeding under prophylactic anticoagulants 3.6% (3.2–3.9%). From these data, it was estimated a postpartum VTE risk ≥ 1% to *possibly* provide a net clinical benefit, and a postpartum VTE risk > 3% to *likely* provide net benefit.

After delivery, the ACCP thus stipulates 3% (i.e., for situations associated with an odds ratio of >10 after vaginal delivery, for which the risk is 0.3%, and >6 after Cesarean delivery, for which the average risk is 0.5%) (21).

In 2018 the American Society of Hematology (ASH) (22) and in 2014 the Society of Obstetrics and Gynecology of Canada (SOGC), estimated it as 1%. The arguments why these societies have chosen a different thrombotic risk threshold are not clearly supported.

In 2015 the RCOG and the ACOG in 2018 (American College of Obstetrics and Gynecology), did not set a threshold but categorized situations into levels of risk, with suggestions per level. It should be noted that, to avoid one VTE during the 6 weeks postpartum for a hundred women with an absolute risk of 3%, and if low molecular weight heparins (LMWHs) are 80% effective, 1,750 injections should be given. For women with an absolute risk of 1%, 5,250 LMWH injections should be given to 125 women to avoid one VTE.

A particular clinical issue is the prevention of recurrence during pregnancy or postpartum in a woman with a personal history of VTE, the strongest individualized preexisting RF for VTE (Table 1B). All women with such a history should be assessed before starting a pregnancy, with information on the risks involved, means of prevention, known data and risk assessment. Postpartum thromboprophylaxis for at least 6 weeks is recommended by almost all the available experts-driven international guidelines, regardless of the mode of occurrence of the prior VTE event. Recommendations are more variable during the pregnancy itself.

In case of an unprovoked or a hormone-related VTE (i.e., associated with an estrogen-containing hormonal contraception or with a prior pregnancy), thromboprophylaxis is recommended during pregnancy. However, the optimum LMWH dosages are still uncertain.

In case of a VTE provoked by a non-hormonal transient RF, and in absence of any other VTE RF, some discrepancies still exist, from thromboprophylaxis only in the third trimester of pregnancy, to postpartum only thromboprophylaxis.

Regarding pharmacological thromboprophylaxis methods before/after childbirth, unfractionated heparins are impractical before, but can be applied after. Although LMWHs are the gold standard, the use of weight-adjusted preventive doses is increasingly suggested on pharmacological grounds, but no work has ever demonstrated its clinical relevance. Pentasaccharide is occasionally used before, but can be used after. Vitamin K antagonists (VKAs) are reserved for women with mechanical heart valves before delivery and can be used afterwards. Direct oral anticoagulants (DOACs) should not be used during pregnancy as they may be teratogenic, nor should they be used afterwards in breastfeeding women. Aspirin crosses the placenta but can be used before and after delivery. However, its effectiveness is highly questionable. Thrombolytics are reserved, before and after, for life-threatening thrombotic situations. The question of LMWH and epidural anesthesia is frequently raised. Local anesthesia techniques should not be applied <12 h after the last preventive injection, and <24 h after the last therapeutic dose injection. LMWH should not be administered within 6 h of epidural anesthesia or after the catheter has been removed. The cannula should not be removed <10–12 h after the most recent injection.

The development of objective Risk Assessment Methods (RAMs) has led some teams to propose scores to guide thromboprophylaxis.

The most notable one is that of the British group, focusing on the assessment of postpartum risk, whose extensive epidemiological studies have led to the publication of a model based on derivation and then validation cohorts (23). This model making it possible to extrapolate the absolute risk for a patient from selected clinical data (23): we have developed a practical online calculable version of this model in our university hospital, http://is.gd/postpartum_risk. The model appears to be more effective than the national guidelines, both British and Swedish, but the area under the ROC curve is still average, slightly over 0.70.

The group in Lyon prospectively described and validated a VTE risk score for pregnancy in 445 heterogeneous women with a history of VTE or with constitutive thrombophilia, accumulating 542 pregnancies (24), the value of the score leads to graduated therapeutic proposals applied to pregnancy, with preventive LMWH systematically prescribed during the postpartum period. The observed incidence of VTE is 8/542: 1.47%, which is at least 10 times higher than the natural incidence of VTE during pregnancy, with no comparison of prophylactic therapeutic modalities.

Another approach, proposed by the Strathège group coordinated in Saint-Etienne (25), was initially based on a national DELPHI method for selecting risk factors and means of prevention (26), constructing a score and proposing progressive prophylactic strategies indexed on that score. Applying a methodological approach before/after use of score-guided prevention in 2,085 pregnant women at risk of VTE or placental vascular complications reduced the incidence of the composite primary outcome [at least one VTE or placental vascular complication: from 19 to 13%, with a reduction of the incidence of DVT: RR 0.30 (0.14–0.67)] without increasing the risk of bleeding (from 3.2 to 4.5%). Placental vascular complications comprised mainly preeclampsia, which relative risk was also reduced: RR 0.52 (0.36–0.75).

These convincing approaches are not yet widely accepted by prophylaxis prescribers, who find them far too complex. However, these methods are full of objective promise and deserve clinical investment. The English algorithm provides an absolute risk value (22) that puts the treatment decision in perspective, particularly in the clinical records. Despite an obvious conflict of interest, we believe that the use of the Saint-Etienne score-guided prophylaxis suggestions has the advantage of having been tested prospectively and shown to be clinically useful (25, 26).

Furthermore, there is no convincing work in clinical biology or laboratory medicine to suggest that the use of functional or genetic laboratory data will make it possible to gain (in terms of relevance and efficiency) in the identification of women who are likely to develop VTE during pregnancy and in the following weeks.

The importance of women's values and preferences with regards to thromboprophylaxis must be discussed and taken in account. A multicenter, international study in women with a history of VTE compared women's choices using a holistic approach in which they were presented all of the relevant information (direct-choice) vs. a personalized decision analysis in which a mathematical model incorporated their preferences and VTE risk to make a treatment recommendation (27). A high degree of discordance between the two decision approaches was observed: 72% of the 72 women for whom the decision model recommended against thromboprophylaxis chose LMWH and 12% of the 51 women for whom the decision model recommended thromboprophylaxis chose not to take LMWH. A cross-sectional, international multicenter study included women with a history of VTE planning pregnancy or being pregnant (28) and determined their values and preferences, and the choices. More women at high risk (defined as women with prior unprovoked VTE or VTE associated with minor transient risk factor with 8 weeks prior to event) than those at low risk of recurrence chose to use LMWH (86 vs. 60%. Given a 16% risk of VTE without prophylaxis, the median threshold reduction in VTE at which women were willing to accept use of LMWH was 3%, interquartile range 1% to 6%. Given the wide variability in patients' values and preferences, patients with similar probabilities of the same consequences will make different choices. Individualized shared decision making is thus needed in the clinical encounter, and weak recommendations for LMWH must be suggested by guideline panels that make necessary the need for individualized shared decision making (28).

## For or Against a Broader Use of Heparin Prophylaxis?

How to best improve thromboprophylaxis around pregnancy remains highly controversial, with strong disagreements between experts and guidelines. Two main practical situations are central to this discussion: pregnant women hospitalized for an antepartum complication (the VTE risk being 17.5 times that of an outpatient pregnancy in the UK study) and Cesarean delivery, both at a high relative risk of VTE events. Some experts do believe that more frequent use of heparin prophylaxis should be encouraged (29) and the best data supporting the safety and efficacy of heparin prophylaxis comes from the UK, with a decline in maternal deaths from VTE in the subsequent 2006–2008 Saving Mothers' Lives triennial report (30), with no associated increased risk of death from hemorrhage being evidenced. Other experts are against a more frequent use of heparins due to costs, lack of evidence and safety concerns [mainly the risk of wound hematomas (31)]. In the ideal situation, a prophylactic regimen based on the conclusions of relevant randomized clinical trials (RCTs) would be recommended. However, we will never have these RCTs because the feasibility of recruitment for such studies is nil, and rare attempts have been failures (32). The analysis of observational data from huge, nationwide registries and prospective cluster clinical trials in which the unit of randomization is not the patient but groups of patients defined, for example, according to the medical ward (hospital) in which they are followed and treated following local prophylactic regimens, might help to qualify/quantify certain clinically-relevant outcomes exploitable for future guidelines.

Going to the Clinicaltrial.gov website, some current studies in pregnant women are found which can draw what to expect in the next future. The NCT01828697 “Highlow” compares low and intermediate dose LMWH to prevent recurrent VTE in pregnancy and results will be soon communicated. The NCT03659708 “Prescot” conducts a medico-economic study to evaluate the efficiency of an innovative strategy integrating the Lyon-VTE-score (24) in the management of pregnant patients with venous thromboembolism risk vs. standard care. The NCT05066867 evaluates LMWH compliance among pregnant and postnatal women undergoing VTE thromboprophylaxis. The NCT01019655 investigate whether heparin is an effective treatment in pregnant women at risk for thrombosis and other pregnancy-associated complications, due to thrombophilia. The NCT02600260 evaluates in-hospital pregnant women through the application of a thromboprophylaxis protocol with risk assessment score. Some new answers will thus be soon available.

## Conclusion

It is therefore clear that, although we have a better understanding of the epidemiology of VTE in pregnancy, its rarity makes its accurate prevention difficult. Conducting therapeutic trials in pregnancy is always a challenge. Making a decision on pharmacological prophylaxis is easy in the most caricatured cases that accumulate risk factors, but remains approximate most of the time. Many points remain unknown: in particular, the precise definition of the populations of women in whom the benefit-risk ratio is acceptable and when to begin prevention during pregnancy and after delivery in the event of obstetric hemorrhage. Also the type of antithrombotic: the use of DOACs after delivery seems to need further exploration, particularly as regards the return home, and perhaps even during breastfeeding as the concentrations of rivaroxaban in milk, for example, do not seem to exceed 10% of that present in maternal blood, and are therefore not clinically relevant. Finally, the dosages and durations need to be better defined.

The heterogeneous expert recommendations show their limits but, as the French humorist Francis Blanche used to say, “*a camel is a horse drawn by a committee of experts.”* The use of “RAMs” (see above) seems to give us great encouragement. Systematic, repetitive assessment of individual thrombotic risk around the time of pregnancy has become compulsory. Teams should finally specify and choose one single common approach whose relevance should be regularly retrospectively evaluated. As randomized trials are unlikely to be conducted here, data from registries and large cohorts of patients are of major help. Finally, we may recall a discussion by Greene-Morton and Minkler (33) on cultural competence and cultural humility in 2020. They stated that believing that one should choose one thing over another would be a poor choice as, in medicine, both concepts have been generated by the professionals' understanding and must consider the biases therein.

## Author's Note

On November 2^nd^, 1907 at the age of 31, the artist Paula Becker (Figure 1), an early figure of German expressionism, gave birth to her daughter Mathilde with great difficulty. After 2 days of labor ending with chloroform, she finally delivered by forceps. Her doctor ordered her to stay in bed. She got up for the first time on November 20^th^, only to collapse and die of a pulmonary embolism.

## Data Availability Statement

The original contributions presented in the study are included in the article/supplementary material, further inquiries can be directed to the corresponding author/s.

## Author Contributions

J-CG developed the idea, supervised, wrote the first draf, and reviewed all subsequent drafts. FG and MC helped in collecting and analyzing the relevant published data. CB and SB reviewed all subsequent drafts. All authors contributed to the article and approved the submitted version.

## Conflict of Interest

The authors declare that the research was conducted in the absence of any commercial or financial relationships that could be construed as a potential conflict of interest.

## Publisher's Note

All claims expressed in this article are solely those of the authors and do not necessarily represent those of their affiliated organizations, or those of the publisher, the editors and the reviewers. Any product that may be evaluated in this article, or claim that may be made by its manufacturer, is not guaranteed or endorsed by the publisher.
